# Barriers and facilitators to guideline for the management of pediatric off-label use of drugs in China: a qualitative descriptive study

**DOI:** 10.1186/s12913-024-10860-0

**Published:** 2024-04-05

**Authors:** Min Meng, Jiale Hu, Xiao Liu, Min Tian, Wenjuan Lei, Enmei Liu, Zhu Han, Qiu Li, Yaolong Chen

**Affiliations:** 1 Chevidence Lab of Child & Adolescent Health, Children’s Hospital of Chongqing Medical University, Chongqing, China; 2 National Clinical Research Center for Child Health and Disorders, Ministry of Education Key Laboratory of Child Development and Disorders, International Science and Technology Cooperation Base of Child Development and Critical Disorders, Children’s Hospital of Chongqing Medical University, Chongqing, China; 3 Chongqing Key Laboratory of Pediatrics, Chongqing, China; 4 Department of Pharmacy, Gansu Provincial Hospital, Lanzhou, China; 5 Department of Nurse Anesthesia, Virginia Commonwealth University, Richmond, USA; 6 School of Public Health, Lanzhou University, Lanzhou, China; 7 College of Pharmacy, Gansu University of Chinese Medicine, Lanzhou, China; 8 Department of Nephrology, Children’s Hospital of Chongqing Medical University, Chongqing, China; 9 Research Unit of Evidence-Based Evaluation and Guidelines, Chinese Academy of Medical Sciences(2021RU017), School of Basic Medical Sciences, Lanzhou University, Lanzhou, China; 10 WHO Collaborating Centre for Guideline Implementation and Knowledge Translation, Lanzhou, China; 11 Lanzhou University GRADE Center, Lanzhou, China

**Keywords:** Pediatric, Off-label use, Qualitative research, Implementation science, China

## Abstract

**Background:**

Despite being a global public health concern, there is a research gap in analyzing implementation strategies for managing off-label drug use in children. This study aims to understand professional health managers’ perspectives on implementing the Guideline in hospitals and determine the Guideline’s implementation facilitators and barriers.

**Methods:**

Pediatric directors, pharmacy directors, and medical department directors from secondary and tertiary hospitals across the country were recruited for online interviews. The interviews were performed between June 27 and August 25, 2022. The Consolidated Framework for Implementation Research (CFIR) was adopted for data collection, data analysis, and findings interpretation to implement interventions across healthcare settings.

**Results:**

Individual interviews were conducted with 28 healthcare professionals from all over the Chinese mainland. Key stakeholders in implementing the Guideline for the Management of Pediatric Off-Label Use of Drugs in China (2021) were interviewed to identify 57 influencing factors, including 27 facilitators, 29 barriers, and one neutral factor, based on the CFIR framework. The study revealed the complexity of the factors influencing managing children’s off-label medication use. A lack of policy incentives was the key obstacle in external settings. The communication barrier between pharmacists and physicians was the most critical internal barrier.

**Conclusion:**

To our knowledge, this study significantly reduces the implementation gap in managing children’s off-label drug use. We provided a reference for the standardized management of children’s off-label use of drugs.

**Supplementary Information:**

The online version contains supplementary material available at 10.1186/s12913-024-10860-0.

## Introduction

Off-label drug use in pediatrics is a global public health issue [1], particularly in China [2, 3]. According to a systematic review, pediatric off-label medicine prescription rates ranged from 22.7% to 51.2% in outpatient settings and 40.48% to 78.96% in hospitalized children in China [4]. However, there are numerous unreasonable examples of off-label drug use in children, posing significant risks to children’s safety [5, 6]. As a result, the Guideline for the Management of Pediatric Off-label Use of Drugs in China (i.e., the Guideline) was developed in 2021 by the Chinese Society of Pediatric Clinical Pharmacology, the Chinese Medical Association, and the National Clinical Research Center for Child Health and Disorders (Children’s Hospital of Chongqing Medical University), in collaboration with the Chinese GRADE Center [7].

However, translating evidence from clinical practice guidelines (i.e., CPG) into practice, also known as implementation [8, 9], is a complex process influenced by various factors such as political and social, the health organizational system, the CPG context, healthcare professionals, and patients [10]. For example, only about half of Chinese healthcare professionals follow the recommendations and understand the clinical practice guidelines, which range from 3 to 86% [9].

To enhance guideline adherence among healthcare professionals, it is necessary to identify the facilitators and barriers to guideline implementation [11]. In addition, theory-based guideline implementation research can assist implementers in avoiding potential pitfalls that may hinder their effectiveness [12]. Consequently, identifying factors that influence the implementation of recommendations, that is, implementation barriers and facilitators [10], is essential for the early clinical translation of guidelines to implement strategies tailored to anticipated barriers [13] and to optimize the implementation of interventions [14].

Off-label use of drugs in children is a complex aspect of clinical practice [15]. Only a small number of studies have demonstrated that the following are obstacles to the management of pediatric off-label use in China: lack of time to offer sources of information and evidence of off-label use, no available expert panel on off-label use, no adverse drug reaction monitoring system, no database of off-label drugs, no ethics council or pharmacy administration committee, difficulties in gaining written agreement from parents or guardians, and absence of a unified regulatory framework [16–18]. In addition, doctors’ awareness prescription of off-label drugs [19–22], their fear of legal repercussions [23], and they are less of informing parents about off-label drugs [21, 24] were obstacles to managing children’s off-label drug use. However, none of the present research is theoretically based on guideline implementation studies and hence may lack systematicity in identifying factors influencing off-label drug use management in children. In addition, implementation strategies for managing pediatric off-label drug use are understudied.

Implementation strategies tailed based on the implementation contextual factors can promote adherence among healthcare professionals [25]. The Consolidated Framework for Implementation Research (CFIR), a well-known implementation science framework, has been extensively used as a framework in recent research on strategies for implementing guidelines, and it has successfully identified the influencing factors for guidelines’ implementation [26–31].Therefore, this study used CFIR for guiding data collection, data analysis, and findings interpretation to implement interventions across healthcare settings and aimed to understand professional health managers’ perspectives on implementing the Guideline in hospitals and determine the Guideline’s implementation facilitators and barriers. Also, the suggestions for implementing the Guideline were created by mapping the identified barriers to the Expert Recommendations for Implementing Change (ERIC) and selecting the appropriate strategies for implementation [26, 32].

## Methods

### Research design

A qualitative descriptive study design was used in this study to understand professional health managers’ perceived barriers and facilitators to implementing the Guideline in hospitals [33]. In the previous study, 896 healthcare professionals from mainland China were invited to complete a questionnaire to rate the urgency and difficulty of implementing each of the 21 recommendations in the Guideline, ranking the recommendations according to combined scores, and selecting the top five of them (See Table 1).

**Table 1:** The content of the five recommendations

Number	Recommendations	Combined scores*
6.2	We suggest that medical institutions embed the evidencebased medicine database of children’s offlabel drugs into the hospital’s electronic medical record system to enhance evidencebased decisionmaking, practice, and management. (3; B)	9117
6.1	We suggest to develop an evidencebased medicine database for offlabel use drug use in children in China. (3; B)	9116
4.1	We suggest using the Benefit and Risk Assessment for Offlabel Use (BRAvO) decisionmaking framework. (2; B)	9011
1.2	Clinicians are recommended to refer to the published guidelines or consensus statements on the specific topic for children. (2; B)	9008
4.2	The risks associated with offlabel use of drugs vary between different age groups of children. It is recommended that clinicians pay particular attention on how to treat neonates and preterm infants. (1; A);Clinicians should consider the child’s age and the associated physiological, growth and development characteristics when prescribing drugs offlabel. (5; C)	9008

* Combined scores were the overall urgency and difficulty scores of the recommendations by 896 healthcare professionals. The urgency of implementation (using a 1–7 Likert scale with a gradual increase in the urgency), and the difficulty of implementation (using a 1–7 Likert scale with a gradual increase in the difficulty)

### Setting and sample

The study was conducted collaboratively by the Clinical Pharmacology Group of the Pediatric Society of the Chinese Medical Association and the National Clinical Research Center for Child Health (Children’s Hospital of Chongqing Medical University). Pediatric directors, pharmacy directors, and medical department directors from secondary and tertiary hospitals across the country were recruited voluntarily through the members’ units for online interviews via Tencent Meeting (https://meeting.tencent.com).

Reading available studies and performing some initial research helped create an interview framework [16, 17, 34–36]. Before the formal interviews started, a pharmacy director was recruited to participate in the pretest, and the interview plan was modified to consider the pretest results. The formal interviews were performed between June 27 and August 25, 2022, and participants were recruited using the convenience sampling approach. All the professionals with at least one year of management experience in pediatric off-label drug use were included. All experts invited to present were encouraged to participate and were given comprehensive information on the study via WeChat. They were instructed to read the Guideline in detail and ask the guideline developers to explain any questions accordingly [7]. Detailed interview times and locations were negotiated after signing an electronic informed consent. The sample size for this investigation was determined based on data coding, data saturation, and study feasibility [37].

### Data collection

A semi-structured interview outline was created, with all questions revolving around the CFIR. The conversation will focus on potential contributing elements and obstacles to the Guideline’s implementation (See Supplementary Material 2). The CFIR framework and pre-interview were used to determine the validity of a structured interview in this qualitative research.

Data collection and analysis were repeated to discover new insights from early interviews that would guide later interviews and data collection [33]. We used Tencent Conferences (https://meeting.tencent.com) for audio recording and Xunfeitingjian Software (https://www.iflyrec.com/) for transcriptions. Each interview was recorded with a particular interviewer label and then transcribed verbatim. All interviewees had the chance to examine the interview recordings to increase credibility and reliability.

### Data analysis

The facilitators and barriers of the Guideline were investigated explicitly in the qualitative content analysis of expert interview data [38]. Both inductive and deductive methods were used to identify facilitators, barriers, and neutral factors [39]. A neutral influence has no positive or negative consequences or both positive and negative consequences but is overall neutral [40]. Meaningful text units, such as sentences, paragraphs, and words, were inductively extracted into coding and then subjected to CFIR framework analysis. These codes were then classified into subcategories and generic categories for further evaluation [41]. Information extraction and coding in Chinese were carried out independently by two researchers (MM and LX), and any discrepancies were resolved through discussion. The final findings were translated into English and further discussed by the research team to enable researcher triangulation and to reach a consensus on the results [42].

### Role of the funding sources

The funder provided support for expert consultation fees and research publication costs. The study’s design and execution were not influenced by the research funding.

## Results

### Characteristics of participants

Individual interviews were conducted with 28 healthcare professionals. The interviews ranged from 21 to 56 min. Half of the participants had a bachelor’s degree, and 60.7% were male. Among the participants, pediatric directors, pharmacy directors, and medical department director were ten, nine, and nine, correspondingly. About 40% of participants had more than 20 years of experience, 27 were in senior positions, and one was in an intermediate position. There were 15 from tertiary hospitals and 13 from secondary hospitals, respectively. Twenty of the professionals interviewed were dissatisfied with the current management of off-label drug use in children. Participants came from all across the Chinese mainland (see Table 2).

**Table 2:** Characteristics of the participating healthcare professionals

Characteristics	Number	Percentage (%)
Gender		
Male	17	60.7
Female	11	39.3
Degree		
Below Bachelor	1	3.6
Bachelor	14	50.0
Master	8	28.6
Doctor	5	17.9
Professional Role		
Head of pediatrics	10	35.7
Head of pharmacist	9	32.1
Medical department director	9	32.1
Work Years		
< 5	2	7.1
6–10	3	10.7
11–15	3	10.7
16–20	9	32.1
> 20	11	39.3
Position ^a^		
Not obtained	0	0.0
Junior	0	0.0
Intermediate	1	3.6
Senior	27	96.4
Hospital Level		
Secondary	13	46.4
Tertiary	15	53.6
Satisfaction degree of Management of Pediatric Off-label Use of Drugs		
Unsatisfied	20	71.4
Mostly satisfied	6	21.4
Indifferent	1	3.6
Satisfied	1	3.6
Region Distribution		
Southwest China	6	21.4
Northwest China	6	21.4
Northeast China	5	17.9
Northeast China	5	17.9
North China	2	7.1
East China	4	14.3

a: Healthcare professionals in China is often classified as “senior”, “intermediate” or “junior” position according to their skill levels and specialization. b: Other types of hospitals, including general hospitals and other specialized hospitals with pediatric clinics

### Identified influencing factors

According to the findings of the interviews, there are 57 factors influencing the implementation of the Guideline in China, including 27 facilitators, 29 barriers, and one neutral factor. These contributing factors were spread throughout 29 constructs in the four CFIR domains studied for the guidelines (see Table 3 and Supplementary Material 1). The most influential factors were found in the internal setting, and the fewest influences were found in the intervention characteristics, which was 24 and ten, respectively. Following the CFIR framework, including intervention characteristics, external setting, internal setting and individual characteristics, we will present the following descriptions of all influential factors.

**Table 3:** Facilitators and barriers to implementation of the guideline

CFIR framework	Facilitating factors	Barrier factors
	Coding	Coding
Intervention characteristics		
Intervention Source	The physician support	A lack of practicality
		Unnecessary clinical practice
Evidence Strength & Quality	The physician trust	/
Relative advantage	More advantageous than comparable existing Chinese similar guidelines	/
Adaptability		A need for context-specific adaptation
Trialability	/	A poor trialability in non-children’s Hospitals
Complexity	/	A poor feasibility in primary Hospitals
		Some complicated recommendations
Cost	/	A need for some cost
Outer setting		
Patient Needs & Resources	Meet children’s treatment needs	/
Cosmopolitanism	Pharmaceutical companies participate in and promote clinical trials	A lack of patients understanding
		pharmaceutical industry off-label promotion
Peer Pressure	The Guangdong Pharmaceutical Society, the Shandong Pharmaceutical Society, and similar guidelines from other countries	
External Policy & Incentives	The Physicians Law of the People’s Republic of China	Non-reimbursement by health insurance
	The occurrence of off-label drug use disputes in children raises concerns in this area	A risk of legal conflicts
	Unique improvement campaigns	A lack of administrative & policy promotion
Inner setting		
Structural Characteristics	Graded management	The low priority of pediatrics in non-children’s hospitals
	A dedicated person to drive	/
	The addition of prescription review rules	/
Networks & Communications	A promotion by societies or associations	The unfavorable social environment and conflict between doctors and patients
	A promotion by medical associations	A lack of communication between pharmacists and clinicians
Culture	Cultural alignment with the hospital	/
Implementation Climate	High urgency	A lack of no priority in comparison to other daily work
	Fitting firmly with the hospital’s management	A lack of personal gain
	Availability of punishments	A low-physician compliance
	Alignment with hospital management goals	Complex management procedures
	A better learning environment	/
Readiness for Implementation	Proper off-label drugs coverage by the hospital	A lack of attention from hospital leadership
	A special team of off-label drug management	A lack of specialized training
	A database of off-label drug use	/
	clinical pharmacists’ support	/
characteristics of individuals		
Knowledge & Beliefs about the Intervention	/	A lack of understanding of the Benefit and Risk Assessment framework
Self-efficacy	An alignment with personal beliefs	/
Individual Stage of Change	Physician confidence	Low titles
	A willingness to promote	A lack of passion and innovation of pharmacists
	A high degree of professional restraint and self-defense of pediatric doctors	A wide range of technical competence
	/	A few physicians’ poor ethical principles
	/	An ignorance of physicians’ management of off-label use drugs
	/	Physicians’ empiricism with drug use

### Intervention characteristics

In seven of the eight constructs in the CFIR domain of intervention characteristics, three facilitators and seven barriers were identified (see Table 3 and Supplementary Material 1). Many experts supported the implementation of the Guideline and praised the quality and strength of the evidence in terms of facilitators. The Guideline’s key relative strengths were the Guideline developed by a pediatric specialty hospital, which was in charge of developing pharmaceuticals for pediatrics, including national interdisciplinary specialists with more impact. It is more advantageous than comparable existing guidelines in China.

The barriers included a lack of practicality, unnecessary clinical practice, a need for context-specific adaptation, poor trialability in non-children’s hospitals, poor feasibility in primary hospitals, some complicated recommendations, and a need for some cost. The participant said, “With or without this guideline, it has little impact on clinical practice; it is just an additional option to consider.” which showed the Guideline is not particularly meaningful. The absence of emergency response capacity, the shortage of pediatricians, and the inability to accurately estimate adverse drug reactions are the key barriers to implementation in primary care facilities. The adaptations to the guidelines that are required to fit the implementing setting include suiting the primary level, renaming off-label drug use to expanded drug use, managing pediatric population subgroups differently (neonates, infants, children, and adolescents), improving process management, and simplifying clinical practice. The management of off-label use of drugs should be implemented for all patients while managing the pediatric population, according to the broad view of non-children’s hospital managers who believe that the pediatric population is too small. Costs that need to be considered include the cost of purchasing, maintaining, and updating the database, the cost of recruiting assessment experts, the cost of legislation, training, and dissemination, as well as the time clinicians must spend managing off-label drugs.

### External setting

In the four constructs of the external setting, a total of 12 influencing factors were included, with five facilitating factors, six barrier factors, and one neutral factor (see Table 3 and Supplementary Material 1). In terms of facilitating factors, the Guideline can meet children’s treatment needs, pharmaceutical companies participate in and promote clinical trials, the Physicians Law of the People’s Republic of China encourages the management of off-label drug use in children, the occurrence of off-label drug use disputes in children raises concerns in this area, and unique improvement campaigns. Neutral influences include the Guangdong Pharmaceutical Society, the Shandong Pharmaceutical Society, and similar guidelines from other countries.

The barriers included a lack of patient understanding, pharmaceutical industry off-label promotion, too many choices, non-reimbursement by health insurance, risk of legal conflicts, and a lack of administrative or policy promotion. Although clinicians may have some authority, they will still have to deal with the problem and risk of off-label use of drugs because patients frequently lack comprehension of their use. " Well-known professionals collect a variety of evidence and then inform the patient of any potential adverse effects,, the parents will claim, ‘I signed the informed permission, but I do not know the medicine and saw the instructions did not include this use. You are a doctor, and you know whether to use it.' if the accident occurs.” In China, the health insurance reimbursement system has a direct impact on clinicians’ treatment behavior, and “there is a big problem with not being reimbursed for any medications that are used off-label. " In addition, the possibility of legal disputes arising from the off-label use of medications in children worries many doctors. A participant said,” After all, there is no particular legislation, and while the Physician Law specifies that off-label drug use is subject to standards and guidelines, there are still risks in practice. " Furthermore, the lack of administrative or policy impetus for the guideline is an essential barrier, “Regarding the current context of hospital medication use in China, the power of professionals is constantly pushed by the force of administration or policy. “

### Internal setting

The 14 structures of the internal setting in CFIR contained the most influencing elements, with 15 facilitators and nine barriers (see Table 3 and Supplementary Material 1). The facilitators included graded management, a dedicated person to drive, the addition of prescription review rules, promotion by societies or associations, promotion by medical associations, cultural alignment with the hospital, high urgency, fitting firmly with the hospital’s management, availability of punishments, alignment with hospital management goals, a better learning environment, proper off-label drug coverage by the hospital, a team of off-label drug management, a database, and clinical pharmacists’ support of off-label drug use. Off-label drugs are not reimbursed by Medicare but are covered by some hospitals. " The hospital will pay for reasonable off-label drugs that are approved by the hospital but are not paid for by health insurance.” Furthermore, many hospitals are prepared to implement off-label management in children, and interview experts believe that clinical pharmacist support can help manage the off-label use of drugs. A participant said, “Our clinical pharmacists are our most important resource for explaining off-label drug use. The combination of clinicians and clinical pharmacists coming together to assess the safety and efficacy of the drugs is particularly good.”

The barriers included the low priority of pediatrics in non-children’s hospitals, the unfavorable social environment, the conflict between clinicians and patients, the lack of communication between pharmacists and clinicians, a lack of priority in comparison to other daily work, a lack of personal gain, low-level physician compliance, complex management procedures, a lack of attention from hospital leadership, and a lack of specialized training. According to many experts, managing pediatric off-label drug use does not prioritize daily work since it is only a small component of rational medication management or daily diagnosis and treatment. A participant said, " Off-label drugs for children are just a minor part of clinical treatment. In the arduous clinical work, I must always prioritize the patient, making off-label drugs impossible to focus on”. Additionally, especially in primary hospitals, there is a lack of specialized training in using off-label medications in children.

### Individual characteristics

In the four constructs of the individual characteristics, a total of 11 influencing factors were included, with four facilitating factors and seven barrier factors (see Table 3 and Supplementary Material 1). The facilitators included an alignment with personal beliefs, physician confidence, a willingness to promote, and a high degree of professional restraint and self-defense of pediatric doctors. The transmission and promotion of guidelines with coworkers, classmates, and some network contacts were mentioned by experts as methods. Furthermore, some interviewers considered pediatricians more self-aware and disciplined than adult physicians.

The barriers included a lack of understanding of the Benefit and Risk Assessment framework, low titles, a lack of passion and innovation on the part of pharmacists, a wide range of technical competence, a few physicians’ poor ethical principles, an ignorance of physicians’ management of off-label use drugs, and a physicians’ empiricism with drug use. Recommendation 4.1’s benefit and risk assessment framework confused many medical professionals. They offered some solutions, such as “I hope to use it as a quantitative adjustment of a scale,” “make it a scoring system,” “make its voice recognizable,” or “make it as intelligent standard operating procedures.” The more considerable barriers are physicians’ empirical use of drugs and a lack of awareness about off-label drug management. “Clinically, there isn’t a clear line between right and wrong, and I think that after the recommendations are put into place, there will be a lot of resistance to changing doctors’ habits if they need to.”

### Role differences

Conflicting views exist among experts on the interaction between clinical pharmacists and physicians. A pharmacist said, “The most challenging component of communicating with clinicians is clinical department chiefs, in particular. Some medical professionals will collect books, manuals, guidelines, and other information to prove their point to you. We must explain that any use not listed in the drug manual is considered off-label, but it may not be irrational. Additionally, you must carefully and exhaustively offer evidence when introducing each form of an off-label drug one at a time. With the medical department, communication is still quite simple.” In contrast, doctors contended that “prescriptions are frequently evaluated by the hospital’s pharmacy department, for example, in the case of incorrect dosage. Then a deduction is required, and much work and time must be spent on fighting and appealing each time.” Clinicians expect pharmacists to devote their time and energy as the driving force behind the off-label use of drugs for children, even though the varied feedback from the roles for communication may be related to the various goals of the different roles for managing off-label drugs for children. A participant said, “Pharmacy is expected by medical departments to offer a catalog or to advance scientific management, but their primary goal is self-preservation and minimizing dangers to clinicians during treatment. Clinicians are also extremely hopeful that pharmacies will become more clinically friendly through constant appeal and standardization, some actions to support the development of a reliable system, and a social environment. However, clinicians might not invest much time or effort in this area.”

### Conflicting influential factors

Some interview experts viewed clinical pharmacists as facilitators, but some believed that they made managing children’s off-label drug use more difficult. “It is appropriate for clinical pharmacists to direct the clinical use of medications because they are more knowledgeable about drug toxicology and adverse effects. But the current situation of over-centralization of clinical pharmacist rights and restriction of clinical use of medications to clinicians, as well as the lack of personal competence of clinical pharmacists, may hinder the rational clinical use of medications, including off-label use in children,” one medical director stated.

Many experts regarded the Law on Doctors of the People's Republic of China as a facilitating factor, but some experts still think there are legal concerns involved in putting the Guidelines into practice. An expert said, “The Physicians’ Law contains 67 items, including four on the use of off-label drugs, which is considerable progress for the management of off-label use of drugs. However, there is no targeted legislation. Clinicians are at higher risk of experiencing adverse side effects from using off-label drugs.” The experts regard the guidelines’ implementation as urgent but not a priority. An interviewer said, “As a result of our current inadequate drug supply and the urgent demand for pediatric medications, experts stressed the urgent necessity to address the issue of off-label prescriptions for children.” However, according to experts, it is not given the highest priority for implementation, primarily due to the busy and complex clinical work and the concern about off-label use of drugs making up a tiny portion of daily work. Additionally, managing children’s off-label drug use is also not a standard component of hospital assessments, and medical staff typically puts the hospital’s assessment requirement first.

## Discussion

According to our knowledge, this is the first study conducted by Chinese guideline developers to tailor the implementation strategy of the guidelines. Key stakeholders in the implementation of the Guideline for the management of pediatric off-label use of drugs in China (2021) were interviewed to identify 57 influencing factors, including 27 facilitators, 29 barriers, and one neutral factor, based on the implementation science CFIR framework and using one-on-one expert in-depth interviews. Based on mapping the critical barriers to the CFIR-ERIC [26, 32], recommendations for implementation strategies were made, such as tailoring strategies, encouraging adaptability, inquiring of national health administrations to promote recommendations, and establishing networks for communication between clinicians and pharmacists. The study revealed the complexity of the factors influencing managing children’s off-label medication use. We will update the Guideline to address the lack of patient awareness, and a lack of policy incentives (non-reimbursement by health insurance and a lack of administrative or policy promotion) were the key obstacles in external settings. The communication barriers between pharmacists and physicians were found to be the most critical internal barriers. Regarding individual characteristics, the main barriers were pharmacists’ varying technical competence and physicians’ empiricism with medication use. Additionally, this study discovered that even though the PRC Physicians Law’s enforcement helped implement and promote the Guideline, it still needs to relieve the issue of legal dangers for medical staff completely. The difference in the barriers to implementing the Guideline for different roles of medical staff is the communication barrier between pharmacists and physicians.

According to this qualitative study, the Guideline was viewed as having less applicability for primary hospitals by many experts. The findings were consistent with a 2017 study on managing children’s off-label drug use, which also found a significant difference between the management of children’s off-label drug use in secondary and tertiary hospitals [17]. In China, each hospital grade has a unique set of medical duties, and the higher the grade, the greater the capacity for treatment [43, 44]. As map CFIR-ERIC suggests, we should tailor strategies [26, 32]. It is advised that guideline developers should take into account the creation of implementation strategies for various hospital grades [14]. Additionally, many experts feel that the Benefit and Risk Assessment Framework in recommendation 4.1 is difficult to comprehend and would like to quantify and improve the framework’s operability to help physicians make speedy and accurate decision-making. Intelligent assisted decision-making technologies have been created globally and deployed in clinical practice [45–47]. Artificial intelligence-based and scientifically sound assisted decision-making systems for children’s off-label drug use to have some shortcomings [45]. As map CFIR-ERIC suggests, we should promote adaptability and suggest researchers should develop a more practical framework for monitoring the use of off-label drugs in children or a scientifically validated off-label medication-assisted decision-making system to make it easy to follow [26, 32].

As our findings show, in China, the lack of policy incentives and Medicare not covering off-label medicine costs are severe barriers to managing off-label drug use in children [48, 49], Belgium [50], the Czech Republic [51], Germany [52], Italy [53], Switzerland [53], the United States [54, 55], Slovakia [55], Greece [5], and Poland [56], were currently capable of paying for certain off-label drugs by general health insurance. As a result, it is proposed that China’s health insurance department consider establishing a national essential specified reimbursement catalog for off-label drugs based on the relevant experience of the countries mentioned above. Also, we find that a lack of administrative & policy promotion is a barrier. Policies are the most influential drivers of medical practice improvement in China. For example, the Chinese Special Rectification Activity on Clinical Antibiotic Use (CSRA), launched in 2011, has been implemented by hospitals and promoted by policy. Numerous studies have demonstrated its rapid and long-term implementation effect [57–59]. Alter incentive/allowance structures, involve executive boards, and build a coalition were mapped by CFIR-ERIC [26, 32]; consequently, the national health administration is called upon to promote implementing off-label drug use management in children.

Although the Law on Doctors of the People's Republic of China was a reasonable basis for off-label use, physicians and hospitals face potential legal risks in practice, according to our research, which may be because of its implementation challenges [59]. According to Chinese Physicians Law, “in special cases where effective therapies are not yet available, a physician may, after obtaining the patient’s explicit informed consent, use a drug that is not stated in the drug’s instructions but has evidence to support its use,” which indicates that there are two conditions for using drugs off-label. First, obtain the patient’s informed consent. Second, there is evidence supported. Clinical challenges exist in obtaining informed permission from parents of children, primarily because of their lack of comprehension of the concept of off-label use of drugs [19, 60] and an increased risk of adverse reaction [60], which is further worsened by the crisis in doctor-patient trust crisis [61]. Additionally, the current inaccessibility of evidence, mainly because of the shortage of locally evidence-based data for pediatrics [62, 63], the shortage of evidence-based specialists [64], and the ignorance of “evidence-based medicine” and its critical databases among doctors both domestically and internationally [65]. As a result, the following two suggestions are recommended: On the one hand, information sharing and disease-specific education [66] can help doctors and patients communicate more effectively. The Guideline’s developers should create patient and public versions of the Guidelines [67, 68] to “translate” the rationale and recommendations into a format that patients and the general public can understand and use, as well as to assist parents of children in understanding the meaning and necessity of off-label drugs in a friendly manner. Parents will have a better grasp of why off-label drug use is necessary. On the other hand, the authors of the recommendations should invite evidence-based specialists to regularly update the “list of common types of pediatric off-label use of drugs, evidence levels, and recommendations” in Recommendation 1.2, making it easy for clinicians to access the evidence-based information regarding the use of drugs off-label in children.

Clinical pharmacists actively contribute to managing off-label drugs in children, as the experts indicated in their interviews [69–72]. However, the study identified communication barriers between pharmacists and physicians, which is consistent with the findings [73]. On the one hand, the idea of the doctor as a leader is ingrained in the medical profession. The power gap between doctors and pharmacists makes doctors seem unapproachable to pharmacists [74, 75]. On the other, most clinical pharmacists in China originally trained as ordinary pharmacists and went on to finish a year of continuing clinical pharmacy education [76, 77]. A need for more clarity of duty and role conflict among clinical pharmacists is frequently the result of shorter training programs and quick duty transitions [76]. The wide range mainly demonstrates this in clinical pharmacist competence [78], which has caused physicians to need more faith in their expertise [73]. In order to improve the communication effectiveness of pediatric off-label use of drug management, it is suggested to investigate appropriate communication strategies and establish networks for communication between doctors and pharmacists according to the CFIR-ERIC map [26, 32]. For instance, physician-pharmacist-patient communication has become more effective and satisfying thanks to the situation-background-assessment-recommendation (SBAR) standardized communication model [79, 80].

### Strengths

To our knowledge, this study significantly reduces the implementation gap in managing children’s off-label drug use. We systematically identified and analyzed the “Guideline for the Management of Pediatric Off-Label Use of Drugs in China” implementation challenges using the CFIR framework and gave suggestions for implementing the Guideline. In this study, we investigated the perspectives of healthcare professionals in various hospital roles on the management of children’s off-label drug use. We provided a reference for the standardized management of children’s off-label use of drugs.

### Limitations

The study also has some limitations. Firstly, only the key stakeholders in the Guideline—the head of pediatrics, the head of the pharmacy, and the medical department director were included in the study, whichmeans that not all influencing factors were identified. Still, since all participants have rich experience in the field and experience managing off-label drug use in children, we believe they are more representative. Second, quotations with codes were translated into English from the expert interviews and data analysis done in Chinese. Although no researchers of the international collaborative team had read the original transcripts, a consensus was reached through an iterative process and triangulation to ensure the objectivity of the data collection and analysis.

### Implications for further research and clinical practice

Planning the implementation of guidelines, including a good fit between implementation strategies, relevant interventions, and contexts, is more complicated and demanding [81]. The findings of this study indicate that future complex interventions for the Guideline will be necessary because of several influencing factors. It is advised that future intervention studies be designed using the new framework for complex interventions, which includes intervention development or identification, feasibility, assessment, and implementation [82]. Partnership, target population-centered, evidence, and theory-based, implementation-based, efficiency-based, stepped or phased, intervention-specific, and combination are currently recommended intervention development and design methodologies [83]. Combining the Chinese implementation settings will be possible concerning numerous implementation strategies, such as workflow and regulation optimization, assessment tool development, resource input, or multidisciplinary collaboration [84]. Consequently, complex interventions may be established to encourage the implementation of guidelines at various levels of the hospital setting. In addition, appropriate process evaluation methods should be adopted to comprehend and better understand the causal mechanisms and contextual factors associated with outcome change [85, 86].

## Conclusion

Despite being a global public health concern, there is a research gap in analyzing implementation strategies for managing off-label drug use in children. In the future, the Guideline will be updated based on facilitators and barriers, and interventions will be created in various settings to advance guidelines’ implementation by guideline developers. Additionally, the findings in this study are regarded as a baseline for comparison with the barriers and facilitators evaluated during and after implementing an intervention to improve the use of off-label drug management strategies.

### Electronic supplementary material

Below is the link to the electronic supplementary material.

Supplementary Material 1

Supplementary Material 2

## Data Availability

To preserve the anonymity of interviewees, the transcribed interviews are not available for sharing. The remaining data generated or analysed during this study are included in this published article and its supplementary information file.
