# Supplementary material for: Barriers and facilitators to guideline for the management of pediatric off-label use of drugs in China: a qualitative descriptive study
Source: BMC Health Serv Res. 2024 Apr 5;24:435. doi: 10.1186/s12913-024-10860-0 (PMC10998389; doi:10.1186/s12913-024-10860-0)
Supplement: Supplementary file 1 — Supplementary Material 1 [file 12913_2024_10860_MOESM1_ESM.docx]

| Supplementary Material 1 Facilitators and barriers with their quotes | | |
| --- | --- | --- |
| CFIR framework | **Coding** | **Quotes** |
| I. Intervention characteristics | | |
| Intervention Source | The physician support | 1. " The five recommendations are generally regarded favorably." 2. " This guideline is very useful in clinical practice, and I feel more welcoming on the inside, which may benefit me in my work." 3. " Excellent work, and I especially agree with recommendation 6.2." 4. " Some off-label medication, for example, is pretty good because it has a more regulated base. It indicates that I'm better for clinicians this way." 5. " You've already taken the first step, which was actually pretty hard but worked well." 6. " To be honest, I think it's incredibly valuable because the medication for children is very particular." 7. " Overall, I think it's fairly nice, and I believe the recommendation will result in a relatively significant improvement." 8. " Overall, it is more pleasant because there were no guidelines in this domain before. It might provide us with recommendations on regulation and management." 9. " Overall, it is still very well-written, balanced, and custodial." 10. " This, in my opinion, is a really positive thing, and it is something that people should become more conscious of in the future." 11. " I believe it is critical and beneficial to our country's current condition." 12. " Your work is valuable and relevant since it uses multiple disciplines to tackle challenges......" 13. " I believe it is appropriate; the child and his or her family believe that there have been times when this medicine has been controversial, such as complaints, and the dosage utilized may simply not be based on......" 14. "Every medicine, whether oral or injectable, has too many limitations for kids, therefore there is plenty of potential for research." 15. " It's a regulatory guideline that has to make us think about it from all perspectives, and we must keep some factors in mind when prescribing off-label medications, which I believe is reasonably helpful." 16. "I believe that the framework of these eight topics is relatively straightforward, based on general management principles, including regulatory bodies, and that the evidence, particularly the fourth one, which focuses on the benefit-risk assessment aspect, is relevant." |
| Evidence Strength & Quality | The physician trust | 1. " The quality of this one is higher than the others." 2. "The guideline has extremely high validity, makes an evidence-based decision on the off-label use of drugs for children, and provides good evidence for clinical quality control." 3. " I believe in the quality and strength of the evidence. " 4. " The evidence-based evidence is sufficient, and I feel more trustworthy." 5. " I have a favorable opinion; it is pretty objective, and it is also very much about saying professionalism and objective." 6. " The level of evidence is objective in all aspects." 7. " The evidence grade is really strong; for example, the FDA or other national evidence is used as a reference. " 8. " Regarding the quality of the evidence, I believe it should be emphasized that children should be more than or equal to adults. So, I support the evidence in the guideline. " 9. " It has sufficient evidence-based evidence, and it is such a level of evidence-based level that it is met above a certain level." 10. "Although I am confident of the quality and force of the evidence, I find the work challenging." |
| Relative Advantages | More benefits than existed | 1. " This one's evidence grade is stronger than the others." 2. " This is more comprehensive than the prior similar ones. " 3. " The validity of the evidence supporting these recommendations is high and helps with decision-making; a children's specialized hospital-led approach is more professional." 4. " Since a comprehensive interdisciplinary team of clinical pharmacists at the national level is involved, I believe this guideline has a different significance and is also well-known." 5. " This guideline has an inherited relationship with the ones that existed before. I think this one should be more standardized and based on many commonalities." 6. " Numerous advantages exist, not the least of which is that the evidence is mainly objective in all other areas. Before, whenever a patient or a member of my department used it, I would ask if I could use it too and then look it up online before suggesting it to him. But now the guideline is trustworthy." 7. " I believe an off-label drug use guideline created by a children's hospital like Chongqing Children's Hospital will be well received if done over a lengthy period of time. " 8. " The management of off-label drug usage is a subject covered by the Shandong Provincial Pharmaceutical Society. However, they don't have a distinct one for children's medications, and a second issue is that they are ambiguous about what management is and how to do a risk assessment." |
| II. Outer setting | | |
| Patient Needs & Resources | Meet children's treatment needs | 1. " This guideline meets the needs of children." 2. "It may meet our patient's needs, or at least serve as a safeguard for the children." 3. "Both the patient and the doctor benefit from it." 4. "Satisfy the possibility of off-label usage of drugs in children." 5. "Overall, advocating for the patient is essentially taking care of the children's needs." 6. "I believe that these recommendations suit our children's demands." 7. "The first one is that these patients need to utilize off-label drugs on some special occasion, so they all meet it because it is a policy directive. The second is that we have put in place a lot of rules and guidelines to make sure that our patients benefit more from using off-label medications while also making sure that they are safe." |
| Cosmopolitanism | Pharmaceutical companies participate in and promote clinical trials | 1. "To promote clinical trials of off-label medications, manufacturers must be able to unite and reach an agreement." 2. "Manufacturers must be able to unite around this and come to an understanding. Like a closed loop to promote off-label drug development. We are willing to add its indications and follow us to locate the evidence." |
| Peer Pressure | The Guangdong Pharmaceutical Society, the Shandong Pharmaceutical Society, and similar guidelines from other countries | 1. "Clinical pharmacists usually look through the Guangdong Province's consensus on off-label drug use and the Beijing Medical Bureau's guidelines on off-label drug use." 2. "The Guangdong Provincial Pharmaceutical Society was the first to suggest using drugs off-label." 3. "Professor Zhang Lingli of West China Hospital is interested in the off-label use of drugs in children. The Guangdong Pharmaceutical Society is the other." 4. "The Guangdong Pharmaceutical Society has been publishing expert consensus on the off-label use of drugs year after year." 5. "The first one is from the Guangdong Provincial Pharmaceutical Society, and other societies from other provinces published some consensus on off-label use of drugs after." 6. "We frequently refer to a catalog published by the Guangdong Pharmaceutical Society, which is pretty powerful." 7. "The off-label use of drugs has published an expert consensus by the Shandong Provincial Pharmaceutical Society." |
| External Policy & Incentives | The Physicians Law of the People's Republic of China | 1. "From a physician's standpoint, there has been a lot of necessary states for off-label drug use since the publication of the Physicians Law." 2. "The Physicians Law refers to four clauses relating to the off-label use of medications. The state, in my opinion, is also paying attention to it. It should be subject to strict regulation, which is essential." 3. The Physicians Law mentions a justification for using off-label medications." 4. According to Article 29 of the Physicians Law, off-label drug use by doctors may be managed at the hospital level. Still, if it causes disputes, such management rules at the hospital cannot be invoked in the law. There is a reasonably significant question. However, regulations and scientifically sound medical evidence of drug use represent a substantial legal advance." 5. "The Physicians Law is essentially a step forward and an endorsement of off-label drug use." |
|  | The occurrence of off-label drug use disputes in children raises concerns in this area | 1. "The pharmacy management committee must pay special attention to the off-label use of drugs unless it comes to the degree of doctor-patient conflict." |
|  | Unique improvement campaigns | 1. "Incidental events, such as medical errors. This analogy may not be appropriate because of a specific event in off-label drug use that resulted in such a severe consequence. Then the pharmacy or medical side brings this up, and the hospital will conduct a special rectification in this case." |
| III. Inner setting | | |
| Structural Characteristics | Graded management | 1. "In the information system, we clinicals should reinforce the graded management of the doctor's prescription authority, so that only doctors with the title of associate senior or higher can prescribe them." |
|  | A dedicated person to drive | 1. "To identify a leading person, leading team, he still may go to the smooth implementation as long as there is a leading person and leading team to promote the recommendations." |
|  | The addition of prescription evaluation rules | 1. "The audit software can retain the off-label pharmaceuticals in the audit rules and is adaptable to the actual situation of the hospital." 2. "If it is acceptable to use an off-label drug, we will add that drug to the audit rules, and the prescription will not be reviewed by the program when the physician prescribes such drugs." |
| Networks & Communications | A promotion by societies or associations | 1. "I believe that all of our medical societies, including our pediatric societies and our specialty committees, should be able to support this." 2. "Our pharmacy society is pushing first so that pharmacists comprehend the guideline for off-label use of drugs. Pharmacists will communicate with pediatricians once they understand it better, and the pediatric specialty committee will campaign for it." 3. "The Society may engage in some advocacy and other related activities, and the promotion ought to be excellent as well." 4. "There are specialized societies that promote what is best, and the society can undoubtedly impact its distribution and promotion." 5. "To promote Chinese medicine, we must first go from the discipline specialty to pediatrics." 6. "Our medical society may continually advocate for academic advancement." 7. "I believe we can promote our academic specialty with the help of our association's and national support." 8. "Pediatric branch or pharmacy meetings are more authoritative since the Chinese Medical Association is pushing for these statements to be strong, and when they promote the statements, clinicians will be more likely to accept them." 9. "The effect of doctors may be noticeable if it comes from the medical association or these physician associations." 10. "Physicians are expected to understand new norms or guidelines, and physician associations lead or promote them." 11. "Medical societies, like the Society of Pediatric Social Medicine, need to promote themselves, and so do other academic groups. They should do this together." 12. "The medical association is more reasonable and appropriate than the pharmacy association because the pharmacy is still on the management side of the content, and the operation and evaluation of clinical doctors are more conduct-based, so the medical association of a promotion should be a very beneficial aspect." 13. "Our society can encourage the use of guidelines because their main purpose is to control clinicians' behavior, such as primary care doctors at all levels, and they often participate in different academic activities." |
|  | A promotion by medical associations | 1. "Similar to using the three-tier vertical management structure of medical treatment combinations, which facilitates the management and supervises performance." |
| Culture | Cultural alignment with the hospital | 1. "Alignment with organizational values." |
| Implementation Climate | High urgency | 1. "Even though I've been a pediatrician for a long time, I need this right away because there aren't enough drugs for kids on the market right now." 2. "Management of pediatric off-label usage of medications is urgently needed because parents occasionally inquire about the specifics." 3. "Medical personnel is greatly needed, i.e., we require this guideline to address the issues in our line of work." 4. "Such a system, including interface with the medical care system in hospitals, is now urgently needed." 5. "The management of children's off-label drug usage is still a necessity." 6. "We want to accomplish this kind of work, and then the equivalent is to serve kids a logical use of drug safety, which is something that both professional and technical employees look forward to very much." 7. "The guide is the urgent need, at least as far as stating that there is currently a loophole, as we need to close the gap." |
|  | Fitting firmly with the hospital's management | 1. "The guidelines are more suited to the hospital's existing management of children's off-label drug usage." 2. "All parts of leadership values are a fit because our current method is also more consistent with the guideline." 3. "The current workflow and practices are still largely consistent with this guideline." |
|  | Availability of punishments | 1. "Drug usage that is unreasonable off-label is subject to punishment." 2. "Only the off-label medications falling within the catalog's scope are appropriate in prescription evaluation, and those outside are considered some other punishment." 3. "We will include such off-label drugs considered unreasonable in the control of medical quality." 4. "Currently, the penalty for managing off-label drug use is linked to prescription evaluation. If the off-label drugs are not filed, or the patient does not sign the informed consent, they will be deemed unqualified once we find them in the prescription evaluation process." |
|  | Alignment with hospital management goals | 1. "Protecting my hospital and clinicians... protecting doctors and patients." 2. "Complying with the hospital's objectives to protect physicians." 3. "Our management's objective is to avoid the risk that gives rise to medical malpractice claims." 4. "Staying in line with hospital objectives." (8 persons) |
|  | A better learning atmosphere | 1. "I will learn about off-label use of drugs in children." 2. "I will consult authoritative experts when I don't understand." 3. "Will participate in discussions and studies." 4. "Will research online or discuss in the department." 5. "Take the initiative to learn and communicate." 6. "Goes online for information and discusses with colleagues." 7. "By professional groups that can discuss together." |
| Readiness for Implementation | Proper off-label drugs coverage by the hospital | 1. " The hospital will pay for reasonable off-label drugs that are approved by the hospital but are not paid for by health insurance. " |
|  | A special team of off-label drug management | 1. "The medical department will organize a validation meeting to determine whether drugs can be used off-label." 2. "The hospital pharmacy management committee implements a related management process." 3. "It relies on the pharmacy management committee for off-label drugs." 4. "A multidisciplinary working group was established for the management of off-label use of drugs." 5. "The Prescription Checker Software has been used by skilled clinical pharmacy staff for prescription evaluation and adding off-label drugs." 6. "The pharmacy management committee needs to approve the off-label drugs, and only after all the specialists have assessed and determined if the drug can be used off-label." 7. "The pharmacy management committee demands that off-label drugs be approved or filed for clinical use." |
|  | A database of off-label drug use | 1. " The hospital has a searchable database." 2. "The hospital has a searchable database of the China Pharmaceutical Reference database named MCDEX." 3. "The hospital has a rational medication database." 4. "The hospital has clinical pharmacy software resources." |
|  | Clinical pharmacists' support | 1. "The clinical pharmacist will improve communication with the clinical department." 2. " If the clinical pharmacist is better aware of this component, they will advocate management. The clinical pharmacist will openly question if this usage is off-label." 3. "The clinical pharmacist supported the evidence that we are more acceptable." 4. "Clinical pharmacy is more concerned about this aspect of work on off-label drug use." 5. "Clinical pharmacists in pediatrics will identify the relevant clinical evidence, and we will discuss it. Clinical pharmacists in pediatrics will guide the use of medications, including labeled substances or contraindications to pairing, which will not be allowed to be used." 6. "To support this excellent organizational structure, pediatrics has a clinical pharmacist." 7. "The clinical pharmacist is extremely supportive of the clinic, who talks with the doctor, after discussion in many aspects, and directs the clinical children's off-label use of pharmaceuticals in real-time." 8. "Need especially excellent pediatric clinical pharmacist-led, step-by-step practice guidelines, such as the first stage of evidence gathering; the second stage of evidence grading; the third stage first from a disease system or several disease systems to promote, or the most clinical concerns about off-label drug use to begin doing." 9. "The clinical pharmacist will determine how he or she is using medications outside of their approved uses and then talk to the doctor about what the evidence shows." 10. "Our resource is the clinical pharmacist, who can train us on off-label drug use, etc.; I think it's beneficial when clinicians and clinical pharmacists collaborate to evaluate the safety and efficacy of these two aspects." |
| IV. Characteristics of individuals | | |
| Self-efficacy | An alignment with personal beliefs | 1. "In keeping with and willing to further their principles." 2. "Values are all in alignment, all to effectively managing of the use of drugs off-label." |
| Sense of self-efficacy | Physician confidence | 1. "I feel confident in the adoption of the guideline since we all anticipate managing off-label drug use in children, especially the neonatal, ICU, and PICU physicians, and we also anticipate that such strong guideline measures will assure the safety of clinical drug use." 2. "Confident, even more, hopeful that it will be implemented by the developer of the Guideline." 3. "Very confident because it benefits both doctors and patients." 4. "The implementation is still more confident, but everybody needs to keep moving forward together." |
|  | A willingness to promote | 1. "Willing to assist people in learning and attempting it independently." 2. "Networking with fellow students and employees is an excellent way to promote." 3. "Willing to proceed directly to the implementation stage." 4. "If it helps me realize that this guidance is necessary for our everyday work, I would be pleased to promote it." 5. "I'm going to fight to have it implemented in my clinical work." 6. "Given that I have worked in this field and this guideline is the best foundation for me, I would take quick action." 7. "It can be adopted right away in our hospital because there is an agreement with the guideline, a good fit with our current processes, and all aspects of leadership values are a good match." |
|  | A high degree of professional restraint and self-defense of pediatric doctors | 1. "Pediatricians are more self-aware and disciplined than others." 2. Pediatricians have a better feeling of self-protection in the process of drug use off-label since they are more rigorous than adult physicians because of the high degree of danger in pediatrics." |

| Barriers | Coding | Quotes |
| --- | --- | --- |
| I. Intervention characteristics | | |
| Intervention Source | A lack of practicality | 1. "The challenge of putting some recommendations into practice." 2. "None of the electronic medical record systems are standardized on a national level. Another problem is that the database is never kept up to date. How can all hospitals in the country work together?" |
|  | Unnecessary clinical practice | 1. " With or without this guideline, it has little impact on clinical practice; at most, it is an additional option to consider." 2. "Concerns about the excessive management of off-label drug use, which impacts the development of new treatments for children." |
| Adaptability | A need for context-specific adaptation | 1. "Can be tailored or modified to meet the medical condition and environment's needs." 2. "Given what we know now and what the public knows, it is suggested that the term "off-label use of drugs" be changed to "extended use of drugs" to avoid future misinformation and public confusion." 3. "Clarify the various pediatric subgroup population management practices." 4. "Stronger process management is recommended." 5. "Expect the management procedure to be simplified to comply with clinical practice." 6. "Different recommendations are required for large tertiary, primary, and provincially associated hospitals." 7. "May not be well suited for primary care hospitals and should be modified to be more relevant at different levels of care." 8. "Not all databases are good for our hospitals because there are many ethnic minorities in our area with different genotypes. It is recommended that our hospitals change the rules for using drugs off-label based on the time of year and nest them into our hospital database." 9. "We hope to enhance the flexibility of the guidelines, whether the catalog or the varied recommendations, as the actual situation is different in each region." 10. "Expanded use sounds more rigorous and publicly acceptable than off-label use." 11. "I believe that layers may be required. For instance, the implementation environment at specialized, secondary, and tertiary hospitals is extremely different." |
| Trialability | A poor trialability in non-children's hospitals | 1. "Pediatrics contributes relatively little to the hospital's earnings. It is recommended that the leadership be more accepting when adults and children are pushed together." 2. "Healthcare facilities place less priority on pediatrics." 3. "The promotion of children's off-label drug use as an addition, primarily by adults, is better received by hospital authorities." 4. "Due to its modest size, the pediatric audience might not be particularly interested. It would be much simpler if you tried it with adults." 5. "If you manage pediatrics with adults, it will be simpler to put into practice. Fewer drugs and individuals are involved when children have been pushed alone, and the sustainability and impact may not be as positive." 6. "Both medical and surgical treatments may have multisystem disorders, and I believe that considering only children is a bit restricting." 7. "As long as it is a non-pediatrics-specialized hospital, pediatrics is a small department. It would be easier to put into practice if we said that the entire population should encourage the management of off-label drug use, with a focus on pediatrics." |
| Complexity | A poor feasibility in primary hospitals | 1. "It would be challenging to reach the primary hospitals, but there may be more implementation challenges at the primary hospitals." 2. "I believe that off-label drug use in the primary hospital should be prohibited because the primary hospital resuscitation measures are somewhat lacking." 3. "In practice, I don't dare to use off-label drugs for children, especially oral or intravenous formulations, because doctors may have to take bigger risks at primary hospitals." 4. "There are fewer pediatricians in primary care, and primary care physicians have poor awareness of off-label prescription use. Furthermore, we cannot definitively trace a patient's adverse reaction to a specific medicine when they experience one." |
|  | Some complicated recommendations | 1. "Having a searchable database is excellent, but I'd like to streamline the database search process so that it takes only five minutes." 2. "The recommendations may be reasonably simple for doctors or pharmacists, but they are a bit hard for hospital administrators." 3. "Some of the recommendations lack clarity." 4. "The benefit and risk assessment framework in recommendation 4.1 is difficult." |
| Cost | Need some cost | 1. "Cost of database purchase, care, and updating." 2. "Formation of assessment experts." 3. "Using advocacy to convince clinicians to adopt these recommendations." 4. "The cost of legal risk is the largest." 5. "Data exchange, maintenance, and collection all have costs." 6. "There are expenses related to the clinical pharmacists' training, the hospital-wide training, and the revision of the review prescription software." 7. "It is the hospital's cost to purchase the necessary database or software." 8. "The real cost is the cost of the software to add cases to the database and the cost of regular updates." 9. "Setting up the database is the most expensive part, followed by the costs of running the business and paying experts to evaluate it." 10. "The main expense is the training cost, the cost of establishing the database, the invitation of a large number of experts to participate, and a significant number of evidence-based studies." 11. "There might be a fee for the database to be included in the prescription audit software or the hospital information system." 12. "There are administration fees, helping the pharmacist get trained, and putting the hospital information system in place." 13. "The benefit and risk assessment framework cost is high, and the database cost is even higher." 14. "The major thing is the economic expense of hospital information technology." 15. "There are a lot of costs to think about, like getting experts together, consulting online, promoting on social media, and paying for the legal department." 16. "Besides the hardware and software expenditures, there may be some costs associated with training, such as the purchase of a database and the monitoring and administration of off-label drug management at a later stage." |
| II. Outer setting | | |
| Cosmopolitanism | A lack of patients understanding | 1. "Patients rarely have the opportunity to study clinical recommendations, so patients do not understand off-label drug use." 2. "Some parents will read the instructions and ask the doctor about the drug before rejecting its use off-label." 3. "Parents will still question the doctor and even make trouble if an adverse event occurs, even if informed consent is written." 4. "It is advised that public awareness of off-label drug use be promoted, so everyone believes that off-label drug use is usual." 5. "It is frequently required to interact with families to convince them that using drugs off-label is reasonable; it is surely not practicable to explain the guidelines to patients, who will not understand such a specialist medical issue." 6. "The parents' lack of understanding of the use of off-label drugs is a huge hurdle." |
|  | Pharmaceutical industry off-label promotion | 1. "I worry that drug companies will wrongly use the recommendations in the guidelines to promote a lot of clinical drugs that are used off-label. This could lead to a problem with adverse promotion." |
| Peer Pressure | Too many choices | 1. "Too much information has been disseminated regarding different off-label uses of drugs. I can not evaluate the merits and quickly distinguish high-quality recommendations." |
| External Policy & Incentives | Non-reimbursement by health insurance | 1. "Medicare does not pay for off-label drugs, and if we prescribe drugs off-label, we risk being penalized when Medicare performs checks, and the hospital will hold the department or person accountable." 2. "There is a big problem with not being reimbursed for any medications that are used off-label." 3. "Only the patient's out-of-pocket expenses are paid for off-label drugs." 4. "Medicare reimburses rigorously according to the instructions. Every time it checks for issues relating to reimbursement for drugs used off-label, it informs the hospital and the doctor to correct the problem, and the hospital may appeal to Medicare. However, it must present a valid justification for the appeal and will likely not be reimbursed." |
|  | A risk of legal conflicts | 1. "Well-known experts take all kinds of evidence and then talk to the patient about what side effects there might be when used. Even if the parents agree, once something happens, the parents will say, 'I signed the informed consent, but I don't understand medicine. You are a doctor. Do you know whether it should be used or not? I just saw that the instructions don't mention this kind of usage." 2. "The court judges don't know medicine and don't understand medical needs. They follow exactly the terms of the law. But I want lawyers to be socially responsible." 3. "As clinicians are more stressed, the first consideration is to let the patient recover soon, but as leaders, they may think more and worry about the legal risk of what we have to bear in case something goes wrong." 4. "After all, there is no targeted legislation, although the physician law says that off-label use of drugs can refer to norms and guidelines, but in reality, there are still risks." 5. "In the event of an adverse event with off-label drug use, there will certainly be a lawsuit." 6. "After off-label use, if a patient does have an adverse reaction, evidence-based evidence does not play a big role in the legal aspect, so there is a lot of trouble for clinical off-label drug users." 7. "Medical malpractice appraisal is recognized, but now there are a lot of disputes that go through the judicial appraisal process, a process that is very unfriendly to doctors." 8. "There is a need for Chinese judges to understand the use of off-label drugs from a legal perspective." 9. "In disputes over civil liability, the hospital is likely responsible." 10. "I think the law is a big obstacle because every doctor is worried about how the court will find the off-label use of drug use behavior and its reasonableness." |
|  | A lack of administrative & policy promotion | 1. "I believe there are too many consensuses or guidelines. The ones that are administratively driven are those that the National Health Commission supervises." 2. "In many circumstances, people believe that if the National Health Commission does not go out and promote it, it will be dismissed as unimportant." 3. "This effort could be put into place more quickly if provincial National Health Commissions at all levels or quality control centers back it up." 4. "The work that is encouraged at the national level and is tied to official performance or evaluation is strong enough." 5. "From the National Health Commission, with administrative powers to push, it may be a little stronger and relatively easier to accomplish." 6. "In the current situation of hospital medication use in China, the power of professionals is always pushed by the force of administration or policy." 7. "It is the level of national support; if the national level does not support, there is no way to fully control this issue." 8. "The hospital cannot prioritize what the National Health Commission does not prioritize." 9. "I believe that the government should be in charge of implementation. The National Medical Products Administration can decide whether to revise drug instructions or add or remove indications." 10. "The National Health Commission or government departments may be able to push the implementation more quickly for the hospital leadership." 11. "The implementation may be long-lasting if the health administration promotes it and steps in, or if the hospital administration promotes it and gets advice from the health administration." 12. "For example, rational drug use and antimicrobial drug management were national promoted. Similar as this, national promotion must be the primary driving factor for the recommendations' implementation." 13. "From the National Health Commission's point of view, the management of off-label drug use can be better promoted, adopted, and put into place, just like the management of antibacterial drugs." 14. "Some national policies or regulations are required, such as those of the Medical Administration and Management Division, to carry out some actions and intervene to promote them." |
| III. Inner setting | | |
| Structural Characteristics | The low priority of pediatrics in non-children's hospitals | 1. "Pediatrics contributes relatively little to the hospital's earnings. It is recommended that the leadership be more accepting when adults and children are pushed together." 2. "As long as it is a non-pediatrics-specialized hospital, pediatrics is a small department. It would be easier to put into practice if we said that the entire population should encourage the management of off-label drug use, with a focus on pediatrics." |
| Networks & Communications | The unfavorable social environment and conflict between doctors and patients | 1. "The medical climate in society ought to have been favorable for doctors to have the courage to approach patients and propose certain potentially dangerous treatment plans in order to best serve their needs. I can only treat a 5 out of 10 patients, which is intrinsically harmful to the patient when I could have given him a 9 out of 10 cure. On the other hand, there are situations where doctors first consider the need to protect themselves." |
|  | A lack of communication between pharmacists and clinicians | 1. "The most time-consuming component of communicating with physicians is the initial step, especially with the clinical department head." 2. "The hospital's pharmacy department frequently reviews prescriptions, and it requires a lot of the doctor's time and effort each time." 3. "Clinical pharmacists provide better pharmaceutical advice in various ways, including selecting antibiotics. Clinical pharmacists, however, are limited to discussing medications from a pharmacy perspective. Additionally, patient care is typically a more involved process, and clinical pharmacists may find it challenging to take the patient's complete medical status into account." 4. "The pharmacy department is involved in the auxiliary clinical process. Many of the opinions and recommendations will be based on clinical advice. If it is in the future in the implementation process and there are issues or some difficulties, it is likely to exist in the clinical and medical communication process." |
| Implementation Climate | A lack of no priority in comparison to other daily work | 1. "The clinical workload is demanding and complex, and off-label drug use in children is only a minor part of the daily work of concern." 2. "This component of off-label drug use probably won't get too much attention, as pediatrics typically uses off-label drugs." 3. "Daily work is usually pretty busy, so it's not possible to say that it's a high priority to finish. However, I think we should still improve how we manage this area." 4. "It will not be specifically aimed at managing the use of drugs off-label, as it is a clinical work that hardly needs to be taken into account." 5. "From an administrative standpoint, there is no assessment in this area. Thus, it is not the performance management indicator that we place the highest focus on in our job." 6. "In the entire pharmacy department, it is not at the top of the list for medication management." |
|  | A lack of personal gain | 1. "There is no compensation for using drugs off-label successfully, but if there is a medical dispute, you are likely to be accountable for the doctor himself." 2. "I'm not likely to gain many advantages from effectively managing drug use off-label." |
|  | A low physician compliance | 1. "Some clinical directors are incredibly well known yet don't comply very well." 2. "Physicians are very busy and may not perform if the process is too time-consuming and cumbersome." 3. "Getting every clinician to be able to embrace the notion or to follow the recommendations may take time, and there are certain obstacles." 4. "The clinic does not necessarily follow or accept the recommendations." |
|  | Complex management procedures | 1. "Some prescription applications take six months to a year to be approved, and if a patient wants it right away, they may have to go to a pharmacy, buy the drug online, or buy it elsewhere at their own expense. Off-label drug use approval is a difficult and time-consuming process." |
| Readiness for Implementation | A lack of attention from hospital leadership | 1. "I believe that physician initiative will primarily drive the application of the recommendations, and the hospital will not employ administrative means to promote." 2. "The hospital is not going to pay attention to the use of off-label drugs except causing any adverse events, such as similar doctor-patient conflicts have caused." 3. "The leadership comments may also rarely focus on this component, as we have no medical controversies or occurrences in this field." 4. "There is no motive for the hospital administration to act to promote." 5. "The pharmacy department is currently the sole one promoting and participating in this area." 6. "As long as using drugs off-label does not result in medical conflicts, leaders tend to pay less attention to it." |
|  | A lack of specialized training | 1. "No training in this area has been provided." 2. "There is an urgent need for community-based education on pediatric medication use." 3. "There are many academic conferences that discuss the use of medications in children, but there is no particular focus on the use of drugs off-label in children. I haven't attended any seminars on this subject." 4. "Training in this field is comparatively scarce." |
| IV. Characteristics of individuals | | |
| Knowledge & Beliefs about the Intervention | A lack of understanding of the Benefit and Risk Assessment framework | 1. "The framework isn't easy for doctors to understand, and it would be best if it worked more easily and objectively as a quantitative scale that can be changed as needed." 2. "Consider recommendation 4.2 to be challenging and poorly understood." 3. "The framework may be accurate, but clinically generally, it's not easy to implement and a little difficult to operate." 4. "The benefit and risk assessment framework is a little complicated. It is overly demanding in comparison to the primary hospitals." 5. "Because of differences in the level of competence of different healthcare organizations, the framework is complex, and the evaluation results will be unequal between healthcare organizations." 6. "The benefit and risk assessment framework needs to be quantified if improved clinic implementation is to be considered. Following quantifying indicators like efficacy and safety, clinicians are then asked to rate the framework before making judgments based on the score. Implementation in the clinic would be easier with this." 7. "It appears hard to try to create this framework into an intelligent standard operating procedure for clinical practice." |
| Individual Stage of Change | Low titles | 1. "Doctors with low titles have a poorer grasp of off-label drug use and are at greater risk." |
|  | A lack of passion and innovation of pharmacists | 1. "Primary hospitals stay busy, but in terms of coping with inspections and daily work, the initiative and enthusiasm of pharmacists are still very lacking." |
|  | A wide range of technical competence | 1. "Some hospitals do not pay attention to the pharmacy department, especially primary hospitals. From the perspective of pharmacists, their professional level is lacking. There is currently a large gap in pharmacists' professional and technical competence." |
|  | A few physicians' poor ethical principles | 1. "Some doctors may seek personal gain in the use of drugs, not to the benefit of patients, and of course, there are black sheep in every profession." |
|  | An ignorance of physicians' management of off-label use drugs | 1. "Clinicians should increase public understanding of the dangers associated with using medications off-label, including any potential adverse effects or risks." 2. "Physicians naturally lack knowledge about how to regulate the use of off-label drugs, and occasionally doctors themselves do not have time to study drug instructions and unintentionally prescribe the off-label drugs thoroughly." |
|  | Physicians' empiricism with drug use | 1. "Doctors think their expertise and experience can eliminate all dangers." 2. "Physicians may be especially concerned about this area given the long-standing medication habits they have developed." 3. "Many things in clinical practice are not right or wrong, and resistance can be very high if a recommendation necessitates a change in a physician's prescription habits." 4. "The use of empirical drugs by pediatricians is usually because they are so subjective and empirical." |
