# Supplementary material for: Barriers and facilitators to guideline for the management of pediatric off-label use of drugs in China: a qualitative descriptive study
Source: BMC Health Serv Res. 2024 Apr 5;24:435. doi: 10.1186/s12913-024-10860-0 (PMC10998389; doi:10.1186/s12913-024-10860-0)
Supplement: Supplementary file 2 — Supplementary Material 2 [file 12913_2024_10860_MOESM2_ESM.docx]

**Supplementary Material 2**

**Interview Guide**

Date:

Location:

Interviewer:

Start time:

End time:

Hello, my name is XX, and I am an interviewer for the implementation study of the Guidelines for **the Management of Pediatric Off-label Use of Drugs in China** (2021). I will be conducting an in-depth interview with you now.

Definition of the Concept

Off-label use refers to the use of drugs beyond the scope of the drug instructions and labels provided by the manufacturer and approved by the National Medical Products Administration of China. Different forms of off-label use include using drugs for unapproved indications, dosage, or frequency, unapproved route of administration, or using an unapproved population (e.g., age group).

(List of materials provided to the interviewer 24h in advance:

1. **Recommendations for the Management of Pediatric Off-label Use of Drugs in China** (2021) in Chinese.
2. **Recommendations for the** Management guideline for the off-label use of medicine in China (2021) in Chinese.
3. "A list of common pediatric off-label use of drugs, evidence levels, and recommendations"(partially) in Recommendation 1.2).

Screening questions

Have you participated in management/work on off-label drug use or rational use of drugs in pediatrics? Participants include clinicians, pharmacists other health workers (medical department, quality control department, health insurance department, hospital administrators, etc.).

1. **General questions**

1. What is your occupation?

2. how many years have you been in this field?

3. Can you briefly describe the management/work on off-label drug use or rational use of drugs in pediatrics?

4. what is the state of off-label drug management in your environment?

5. what is your highest level of education?

1. **Questions relating to pediatric off-label drug use**

-How do you think about the management/work on pediatric off-label drug use or rational use of drugs in your hospital?

Tips.

Understanding of the importance of the management of pediatric off-label drug use.

Role's assumption in the management of pediatric off-label drug use.

Different roles assume actual content.

Satisfaction with current management status.

-Do you feel confident in your hospital's (region's) ability to promote the implementation of the Guidelines? [If there is a different opinion on the guideline recommendations, discuss them before the next step].

1. **Questions on barriers and facilitators**

-What are your facilitators in managing pediatric off-label drug use?

-What are the barriers to managing pediatric off-label drug use?

1. **Concluding questions**

Thank you for participating in this interview and for your thoughtful answers to all the questions.

Are there any questions we missed in our interview today?

Do you feel we have missed any questions in response to our topic today?

Are there any suggestions for implementing the other recommendations of this guideline?

| Consolidated Framework for Implementation Research | | |
| --- | --- | --- |
| **Topic/Description** | | Brief Description |
| 1. **INTERVENTION CHARACTERISTICS** | |  |
| A | Intervention Source | What do you think about these four recommendations in the guideline? |
| B | Evidence Strength & Quality | What do you think about the quality of the evidence and efficacy of these 4 recommendations in the guideline.? |
| C | Relative advantage | Which do you think is more advantageous, this guideline or the Management guideline for the off-label use of medicine in China (2021)?  Alternatively, would this Guide be more advantageous than in managing pediatric off-label drug use that you have experienced? Why? |
| D | Adaptability | What adaptations are needed to use these four recommendations in your hospital? |
| E | Trialability | Are you likely to attempt these four recommendations in the guideline? |
| F | Complexity | How complicated are these recommendations? |
| G | Design Quality and Packaging | / |
| H | Cost | What cost might be considered if you and your colleagues implement the recommendations? |
| II. OUTER SETTING | |  |
| A | Patient Needs & Resources | How will these recommendations meet the needs of pediatrics? |
| B | Cosmopolitanism | What information on pediatric off-label drug management has been shared with people outside your department? |
| C | Peer Pressure | Are there any other organizations or groups, or individuals that you know of that promote the management of pediatric off-label drug use? |
| D | External Policies | What national/regional/sectoral performance measures, policies, laws, or guidelines influence the use of these recommendations? |
| E | Incentives | What economic or other incentives would influence us to implement these recommendations? |
| III. INNER SETTING | |  |
| A | Structural Characteristics | How do you think your hospital or department can impact the implementation of these recommendations? |
| B | Organizational structure | How do you think your organization's structure may influence your implementation of these recommendations in practice? |
| C | Networks | How do you think your social networks influence your implementation of these recommendations in practice? |
| D | Communications | How do you think the nature and quality of official and informal communication within your organization or department influence your implementation of these recommendations in practice? |
| E | Culture | How do you think the organizational or departmental culture (beliefs, values, and worth) influences the implementation of these recommendations in practice?  How do these recommendations fit the organization's or department's values and standards? |
| F | Implementation Climate |  |
| 1 | Tension for Change | Is there a great need to manage pediatric off-label drug use in your workplace or department? Why? |
| 2 | Compatibility | How do these recommendations fit with the organization or department's current work procedures or practices? |
| 3 | Relative Priority | How will you juggle other works as you practice these recommendations?  Compared to other important works, how important do you think the management of pediatric off-label drugs is in clinical practice? |
| 4 | Organizational Incentives & Rewards | Are there any special recognitions or awards related to pediatric off-label drugs in your organization or department? What is it? |
| 5 | Goals and Feedback | Has your organization or department set goals for managing pediatric off-label drugs?  Have you received any feedback on the management of pediatric off-label drugs?  Are these recommendations in line with the organizational/professional goals for your work? |
| 6 | Learning Climate | What would you do if there is confusion in your clinical practice/work on managing pediatric off-label drugs? (Learning, communication) |
| G | Readiness for Implementation | (In the future) |
| 1 | Leadership Engagement | What recognition or endorsement have you seen or heard from leaders?  Up to now, how involved has your organization or department been in managing pediatric off-label drugs?  What kind of support or actions do you expect from your organization's or department's leaders to help facilitate these recommendations? |
| 2 | Available Resources | Do you have sufficient resources to facilitate these recommendations in your work?  Do you have sufficient resources to facilitate these recommendations in your work?  What are the important/necessary resources (e.g., databases, toolkits) to help you drive these recommendations in your practice? (What are the conditions for implementing these recommendations?) |
| 3 | Access to knowledge and information | What training have you received related to pediatric off-label drugs? [Note: Select all that apply].   1. Lectures; b. seminars; c. problem-based learning; d. case-based learning, e., simulation training; f. other. e. Never received any relevant training. |
| IV. CHARACTERISTICS OF INDIVIDUALS | |  |
| A | Knowledge about the Intervention | Which of the recommendations seemed difficult for you to understand? |
| B | Beliefs about the Intervention | Do these recommendations match your values? |
| C | Self-efficacy | Are you confident in implementing these recommendations successfully? |
| D | Individual Stage of Change | Have you prepared to implement these recommendations? (Anticipated)  ——Knowledge stage (pre-thinking): understanding of key aspects of these recommendations.  ——Persuasion stage (deeper thinking): agree with these recommendations, discuss them with others, accept the implementation, and have a positive perspective.  ——Decision stage (preparation): intend to seek more information and try out.  ——Implementation phase (action): get more information, implement these recommendations, and keep moving forward  ——Confirmation phase (maintenance): benefits are recognized, recommendations are routinely incorporated, and promoted to others. |
| E | Individual Identification with Organization | Would you like to help others? Are you always aware of the process? |
| F | Other Personal Attributes | Have you had any personal experience? Abilities? Motivation? Do you eager to try something new? |
